# Spatial analysis of the association between convenience store density and the prevalence of obesity and hypertension in South Korea

**DOI:** 10.3389/fpubh.2026.1857479

**Published:** 2026-07-22

**Authors:** Ahyoung Yun, Hyein Jung, Goeun Jung, Byungmi Kim, Yoonjoo Choi

**Affiliations:** 1 Division of Cancer Prevention, National Cancer Control Institute, National Cancer Center, Goyang-si, Gyeonggi-do, Republic of Korea; 2 Department of Public Health & AI, Graduate School of Cancer Science and Policy, National Cancer Center, Goyang-si, Gyeonggi-do, Republic of Korea

**Keywords:** convenience store, food environment, geographic information system, hypertension, obesity, spatial analysis

## Abstract

**Background:**

The growing demand for quick and convenient meals has altered eating behaviors and contributed to the rise in convenience stores in South Korea. Convenience stores often provide energy-dense, nutrient-poor foods, which may contribute to unhealthy eating behaviors and increase chronic disease risk. This study aims to examine the association of convenience store density with the prevalence of chronic diseases across 250 districts in South Korea.

**Methods:**

District-level data on the prevalence of obesity and hypertension, as well as covariates, were obtained from the 2022 Korea Community Health Survey. Data on convenience stores were sourced from the 2022 Census on Establishments. Moran’s *I* and LISA analyses were conducted to assess and identify spatial autocorrelation and clustering patterns in obesity and hypertension prevalence across South Korea. Additionally, ordinary least squares (OLS) regression and spatial error model (SEM) were employed to examine the association between convenience store density and disease prevalence.

**Results:**

The results of the OLS model indicated that convenience store density was associated with both obesity and hypertension in South Korea; however, only its association with hypertension was found to be significant, after adjusting for spatial autocorrelation using the SEM. The prevalence of hypertension increased by 0.968% for each additional convenience store per 1,000 people.

**Conclusion:**

Convenience store density was significantly associated with hypertension, even after accounting for spatial autocorrelation. These findings highlight the need for further research and policy interventions to mitigate the health risks associated with convenience store density in influencing hypertension and obesity.

## 1 Introduction

Obesity and hypertension are major noncommunicable disease risk factors and remain important public health concerns worldwide (1). Both conditions are influenced by individual behaviors and broader environmental contexts, including smoking, alcohol consumption, physical inactivity, and dietary habits (2). In particular, frequent consumption of processed foods high in calories, sodium, fats, and sugars has been recognized as an important contributor to obesity and hypertension (3–5). Accordingly, increasing attention has been paid to the food environment as an important determinant of dietary behaviors and chronic disease risk (6–9). Neighborhood characteristics may shape food access, food choices, and long-term health outcomes, suggesting that environmental context should be considered alongside individual risk factors in chronic disease prevention.

In South Korea, rapid socio-demographic shifts have altered everyday eating pat-terns. The rise in single-person households, increased labor market participation, and expansion of the food service industry have collectively fostered a heavy reliance on dining out, processed foods, and ready-to-eat meals (10, 11). Within this context, convenience stores have emerged as a dominant food source, moving beyond their traditional role as supplementary retail outlets. Their high spatial accessibility, long operation hours, and wide range of affordable processed foods make them particularly relevant to busy urban populations (12). According to Statistics Korea, the number of convenience stores increased from 43,975 in 2019 to 57,617 in 2022, representing a 31% increase (13). These trends suggest that convenience stores are no longer merely supplementary retail outlets, but increasingly function as part of the everyday food environment in Korea.

This environmental shift carries profound health implications. Foods sold in convenience stores are often ultra-processed, characterized by high energy density and excessive sodium content, while being relatively low in essential nutrients (11, 12, 14). Previous studies have reported that areas with a higher density of convenience stores are associated with greater purchases of ultra-processed foods (15), and that greater accessibility to convenience stores is associated with unhealthy dietary habits (16). In particular, a higher intake of ultra-processed foods is associated with poor overall dietary quality, characterized by displacement of fresh and minimally processed foods and excessive intake of sodium, saturated fats, and added sugars (17). Such imbalanced dietary patterns may lead to excessive energy intake, excessive sodium intake, and metabolic dysregulation which are key mechanisms underlying obesity and hypertension (18, 19). Accordingly, these mechanisms suggest that convenience store environments may influence residents’ dietary intake patterns and nutritional quality, which may in turn increase the risk of chronic diseases. Prior studies have found that higher accessibility and density of convenience stores are positively associated with obesity (20–22), while the presence and frequent use of convenience stores have also been linked to hypertension prevalence (23, 24). However, findings have not always been consistent across study settings and outcomes.

Despite the rapid growth of convenience stores in South Korea, research examining their association with chronic diseases remains limited, particularly from a spatial perspective. Because convenience store density and chronic disease prevalence may be geographically clustered across neighboring districts, spatial dependence should be considered when examining their associations. Therefore, this study aimed to examine the spatial distribution of convenience store density, obesity, and hypertension across 250 districts in South Korea and to assess their associations using GIS-based spatial analysis.

## 2 Methods

### 2.1 Study design and data collection

This study was designed to investigate the association between convenience store density and chronic disease prevalence among adults in the 250 cities, counties, and districts in South Korea. To examine regional (district level) characteristics, we employed publicly available secondary national datasets, including the 2022 Korea Community Health Survey (KCHS) (25), 2022 Census on Establishments (26), and resident registration population data (27).

KCHS is a nationwide survey conducted by the Korea Disease Control and Prevention Agency (KDCA) to generate health statistics at the city, county, and district levels (25). To ensure an accurate representation of the Korean population, the survey employs a multistage, stratified, random sampling method, incorporating probability proportional cluster sampling (28). A total of 231,785 respondents participated in the 2022 KCHS. Participants aged ≤19 y or who lacked data on body mass index (BMI), hypertension diagnosis, or covariates were excluded from the study. After exclusion, 227,819 participants were analyzed at the district level. The number of convenience stores, obtained from the 2022 Census on Establishments, was used to calculate the convenience density relative to the population in each district.

### 2.2 Dependent variable: obesity and hypertension prevalence

The prevalence of obesity and hypertension at the city, county, and district levels in South Korea was calculated using data from the 2022 KCHS. The prevalence of obesity was calculated based on the proportion of participants with a BMI ≥ 25 kg/m^2^ according to the criteria established by the Korean Society for the Study of Obesity (29). The prevalence of hypertension was calculated based on the proportion of participants with a clinical diagnosis of hypertension.

### 2.3 Independent variable: convenience store density

The number of convenience stores at the district level in South Korea was obtained from the 2022 Census on Establishments data, and the district-level population in South Korea was determined from the 2022 Resident Registration data. Thereafter, the number of convenience stores per 1,000 people was calculated to determine the convenience store density at the district level.

### 2.4 Covariates

Covariates were calculated using data from the 2022 KCHS, with categorical variables aggregated as district-level proportions. Sociodemographic covariates included the proportions of adults aged 20–39 years, men, single-person households, those with a high school education or lower, unemployed individuals, and those with a monthly household income <3 million South Korean won. Lifestyle and health-related covariates included current drinking (≥once/month in the past year), current smoking, physical inactivity (no moderate-intensity physical activity for ≥30 min on ≥5 days/week or vigorous-intensity activity for ≥20 min on ≥3 days/week), weight-control attempts in the past year, high perceived stress (“very much” or “a lot”), and poor self-rated health (“fair,” “poor,” or “very poor”).

The hypothesized relationships among convenience store density, covariates, dietary behaviors, and obesity and hypertension prevalence are illustrated in a directed acyclic graph (DAG) (Supplementary Figure S1).

### 2.5 Data analysis

The KCHS data of 250 cities, counties, and districts was extracted using a complex sampling design. The data was analyzed using the PROC SURVEY procedure in the SAS v9.4 software (SAS Institute Inc., NC, USA), taking into account strata, clusters, and weights. The variables and covariates were combined as shapefiles. Thereafter, the chronic disease prevalence and convenience store density were mapped to visualize their spatial distribution using QGIS™ v3.10.^1^

Moran’s *I* was used to measure spatial autocorrelation across South Korea to determine similarities in the spatial patterns of obesity and hypertension prevalence. Moran’s *I* statistic was analyzed using the GeoDa software v1.22.0.8. Local indicators of spatial autocorrelation (LISA) analysis were performed to identify clusters of spatial autocorrelation in regions with similar characteristics. Based on Moran’s *I* statistic, the LISA plots were categorized into five clusters: High–High (regions with high values surrounded by other high-value regions), Low–Low (regions with low values surrounded by other low-value regions), High-Low, Low-High, and Not Significant.

A linear regression model using an ordinary least squares (OLS) method was used to analyze the association between convenience store density and obesity and hypertension prevalence in South Korea. OLS was initially used as a baseline global regression model to examine the overall association between convenience store density and chronic disease prevalence. Spatial autocorrelation in the OLS residuals was assessed using Moran’s *I* statistics. As the OLS model does not account for spatial autocorrelation, spatial dependence was assessed using Moran’s *I* statistics and Lagrange Multiplier diagnostics. Based on the diagnostic results, a spatial error model (SEM) was used to address this concern in the residuals. Among the two models, the one with a lower Akaike information criterion (AIC) value was considered to be the better-fitting model. Multicollinearity among the variables was assessed using the variance inflation factor, and all the values were <10. These analyses were performed using the spdep v1.3 and spatialreg v1.3 packages in the R v4.4.1 software.

A sensitivity analysis using a reduced model was conducted, and the results are presented in Supplementary Table S1.

## 3 Results

### 3.1 Descriptive statistics

Descriptive statistics of the study variables across 250 cities, counties, and districts in South Korea are presented in Table 1. The mean convenience store density at the district level was 1.12 per 1,000 residents. The mean prevalence of obesity and hypertension at the district level was 30.98 and 22.76%, respectively. The district-level proportions of the sociodemographic and lifestyle and health-related factors of the participants are summarized in Table 1.

**Table 1:** Descriptive statistics of the study variables in South Korea.

Variables	Mean	SD
Convenience store density	1.12	0.41
Obesity prevalence (%)	30.98	0.13
Hypertension prevalence (%)	22.76	0.12
20–30 age group (%)	31.35	0.15
Men (%)	49.71	0.10
Single-person households (%)	15.31	0.12
High school graduate or lower (%)	55.21	0.16
Unemployment (%)	35.61	0.14
Lowest income level (%)	40.57	0.20
Current drinking (%)	54.10	0.15
Current smoking (%)	17.84	0.11
Physical inactivity (%)	77.70	0.12
Weight control attempt (%)	63.44	0.14
Individuals reporting subjective stress (%)	22.55	0.12
Subjectively unhealthy individuals (%)	54.08	0.15

Lowest income level refers to individuals with a monthly household income below 3 million Korean won. Physical inactivity is defined as engaging in vigorous physical activity for <20 min/d for <3 d/week or moderate physical activity for <30 min/d for <5 d/week.

SD, standard deviation.

### 3.2 Spatial distribution of convenience store density and obesity and hypertension prevalence

**Figure 1:**
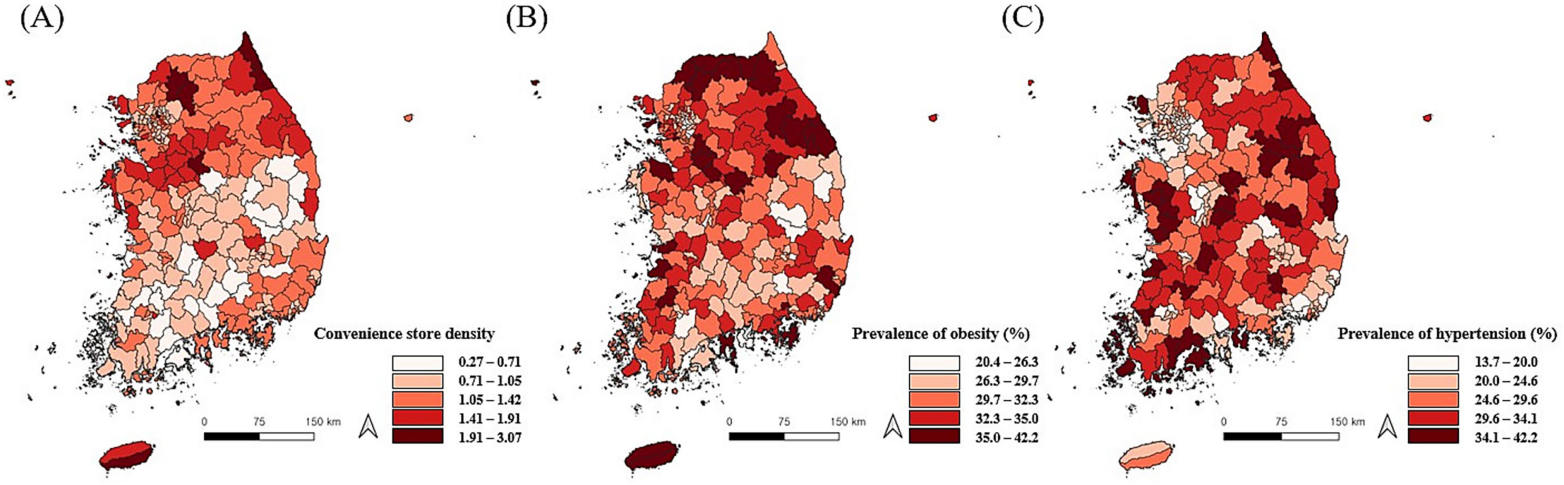
Distribution of convenience store density and chronic diseases prevalence at the district level. **(A)** Convenience store density, **(B)** obesity prevalence, and **(C)** hypertension prevalence. The maps were classified into five categories using the Jenks natural breaks classification method.

Although the spatial analysis was conducted at the district level (*n* = 250), for clarity and interpretability, the results are described based on the 17 broader administrative regions (i.e., provinces and metropolitan cities). A map illustrating these administrative regions is provided in Supplementary Figure S2. to support geographical understanding. The spatial distributions of convenience store density and obesity and hypertension prevalence at the district level were visualized using maps (Figure 1). The convenience store density per 1,000 residents was 0.27–3.07, while the prevalence of obesity and hypertension was 20.4–42.2% and 13.7–42.2%, respectively. Convenience store density was higher in the northern regions than in the southern regions, with the exception of Jeju. The prevalence of obesity was higher in most areas of Gangwon-do, Jeju-do, as well as certain regions of Gyeonggi-do, Chungcheongbuk-do, and Ulsan, while it was relatively lower in parts of Gyeongsangbuk-do and Seoul. Meanwhile, the prevalence of hypertension was elevated in certain areas of Gangwon-do, Gyeongsangbuk-do, Chungcheong-do, and Jeolla-do but lower in Seoul, Sejong, Gwangju, and parts of Gyeonggi-do.

**Figure 2:**
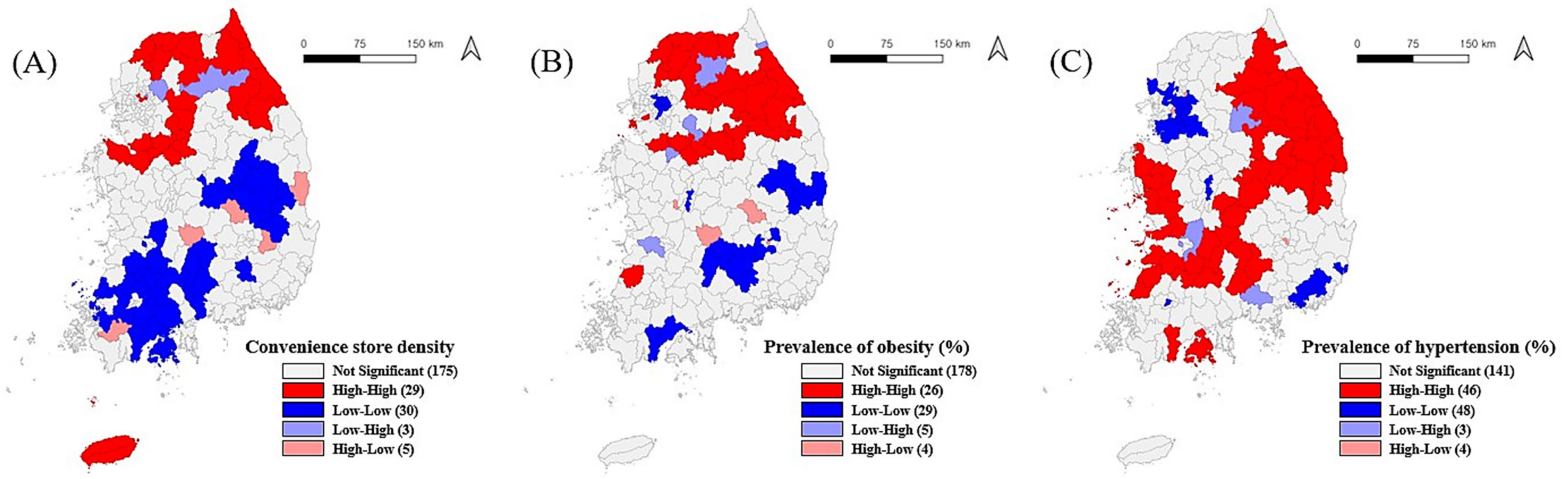
Local Moran’s *I* of convenience store density and chronic diseases prevalence at the district level. **(A)** Convenience store density, **(B)** obesity prevalence, and **(C)** hypertension prevalence.

Moran’s *I* statistic at the district level indicated the presence of global clustering in convenience store density (*I* = 0.401, *p* < 0.001), obesity prevalence (*I* = 0.345, *p* < 0.001), and hypertension prevalence (*I* = 0.534, *p* < 0.001) (Figure 2), suggesting that these factors are not randomly distributed across districts in South Korea. The most areas of Jeju and northern regions showed clusters of high convenience store density, while the southern regions showed clusters of low convenience store density. The northeastern regions of Gyeonggi-do, Gangwon-do, and northern Chungcheongbuk-do showed clusters of high obesity prevalence, while Gyeongsangbuk-do, Gyeongsangnam-do, Daegu, and parts of Jeollanam-do showed clusters of low obesity prevalence. Lastly, Gangwon-do, northern Gyeongsangbuk-do, Chungcheongnam-do, and Jeollabuk-do showed clusters of high hypertension prevalence, while the metropolitan areas and southern Gyeongsangnam-do showed clusters of low hypertension prevalence.

### 3.3 OLS and SEM analyses of convenience store density and obesity and hypertension prevalence

The association between convenience store density and obesity and hypertension prevalence was analyzed using OLS and SEM analyses (Table 2). The results of the OLS analysis indicated that, after adjusting for other variables, convenience store density was significantly and positively correlated with both obesity and hypertension prevalence. The Moran’s *I* values for the two OLS models suggested the presence of spatial autocorrelation in the error terms, necessitating the use of spatial regression analysis. After adjusting for spatial autocorrelation, using the SEM, convenience store density was no longer significantly associated with obesity, whereas a significant association remained for hypertension. In contrast, the model showed that hypertension prevalence increased by 0.968% for every additional convenience store per 1,000 people. Lastly, both obesity and hypertension prevalence showed lower AIC values in the SEM compared to the OLS model, indicating that the SEM provided a better model fit.

**Table 2:** Results of OLS model and SEM on obesity and hypertension prevalence.

Variables	Obesity prevalence		Hypertension prevalence	
	OLS^a^	SEM	OLS	SEM
Convenience store density	1.053 (0.442)^*^	0.790 (0.452)	2.098 (0.443)^***^	0.960 (0.441)^*^
20–30 age group (%)	0.076 (0.058)	0.104 (0.058)	−0.417 (0.058)^***^	−0.450 (0.056)^***^
Men (%)	0.382 (0.136)^**^	0.332 (0.130)*	−0.216 (0.136)	−0.102 (0.121)
Single-person households (%)	−0.063 (0.048)	−0.051 (0.047)	0.001 (0.048)	0.020 (0.045)
High school graduate or lower (%)	0.128 (0.037)^***^	0.129 (0.036)^***^	0.218 (0.037)^***^	0.182 (0.035)^***^
Unemployment (%)	−0.077 (0.038)^*^	−0.086 (0.037)^*^	−0.074 (0.038)	−0.063 (0.035)
Lowest income level (%)	0.022 (0.030)	0.021 (0.029)	0.029 (0.030)	0.053 (0.027)
Current drinking (%)	0.088 (0.050)	0.081 (0.051)	−0.076 (0.050)	−0.056 (0.049)
Current smoking (%)	0.205 (0.085)^*^	0.168 (0.083)^*^	−0.182 (0.085)^*^	−0.131 (0.079)
Physical inactivity (%)	0.029 (0.026)	0.030 (0.025)	−0.019 (0.026)	−0.036 (0.023)
Weight control attempt (%)	0.062 (0.033)	0.061 (0.032)	−0.010 (0.033)	0.020 (0.029)
Individuals reporting subjective stress (%)	0.051 (0.060)	0.018 (0.056)	0.166 (0.060)^**^	0.065 (0.052)
Subjectively unhealthy individuals (%)	0.022 (0.039)	0.033 (0.038)	−0.028 (0.039)	0.030 (0.035)
Lambda		0.2961^**^		0.5368^***^
Moran’s *I*	0.1149^**^	−0.0084	0.2205^***^	−0.0503
*R* ^2^	0.4459		0.8500	
Adjusted *R*^2^	0.4154		0.8418	
AIC	1175.1	1168.2	1176.7	1143.1

a Values are presented as coefficient (standard errors).

*p < 0.05, **p < 0.01, ***p < 0.001.

Lambda (*λ*) in the spatial error model (SEM) indicates the degree of spatial autocorrelation in the error terms. A significant and positive *λ* suggests that residuals in one region are spatially correlated with those in neighboring regions, highlighting the presence of spatial dependency that must be accounted for to avoid biased estimates.

Lowest income level refers to individuals with a monthly household income below 3 million Korean won. Physical inactivity is defined as engaging in vigorous physical activity for <20 min/d for <3 d/week or moderate physical activity for <30 min/d for <5 d/week.

OLS, ordinary least squares; SEM, spatial error model; AIC, Akaike information criterion.

## 4 Discussion

This study utilized spatial analysis methods to examine the distribution and regional clustering of convenience store density and obesity and hypertension prevalence in South Korea. Additionally, it investigated the association between convenience store density and the prevalence of the two chronic diseases in South Korean adults at the district level. The OLS analysis revealed a significant positive association between convenience store density and the prevalence of both obesity and hypertension. However, after accounting for spatial autocorrelation, the association between convenience store density and obesity prevalence became statistically insignificant, while that between convenience store density and hypertension prevalence remained significant. These findings suggest that a higher convenience store density may be associated with higher hypertension prevalence at the district level.

The consumption of convenience store foods has increased in South Korea, due to economic, cultural, and environmental factors, leading to the further emergence of convenience stores (30, 31). Since 2016, South Korea has been found to have the highest convenience store density per capita, exceeding that of Japan and Taiwan (32). In addition, there has been a steady increase in the purchase of home meal replacements, instant foods, processed foods, and ready-to-eat meals from convenience stores (30, 33). Notably, South Korean university students have been reported to frequent convenience stores several times a week for meals (14).

Many foods sold in convenience stores are high in sodium, calories, and sugar, while being relatively low in nutritional quality (30, 32). Previous studies have shown that a higher density of convenience stores is associated with increased purchases of ultra-processed foods and sugar-sweetened beverages (15), whereas greater accessibility to convenience stores has been linked to lower diet quality and reduced consumption of healthy foods (16, 34). Taken together, these findings suggest that convenience store environments may shape residents’ dietary intake quality by increasing reliance on nutritionally poor foods and beverages. Such dietary patterns may represent an important pathway linking convenience store density to chronic disease risk.

In this study, convenience store density remained significantly associated with hypertension after adjustment for spatial autocorrelation, whereas its association with obesity became statistically insignificant. One possible explanation is that hypertension may be more directly linked to aspects of the convenience store food environment, particularly high sodium intake, as well as easy access to alcohol and tobacco products. By contrast, obesity is likely to reflect a broader and more cumulative set of behavioral, environmental, and socioeconomic influences.

Our OLS regression results indicate a positive association between convenience store density and the prevalence of both obesity and hypertension, consistent with previous research. A study in Brazil found that living near convenience stores is associated with a higher BMI and a greater prevalence of overweight and obesity (21). Similarly, studies reported a positive association between convenience store density and higher BMI in adults in Mexico, as well as an increased obesity rate among adults in the United States (20, 22). Although there is limited research on the association between hypertension and convenience stores, existing studies indicate that the presence of convenience stores can increase hypertension prevalence in that region (23). Additionally, frequent use of convenience stores has been found to be associated with hypertension prevalence in patients with type 2 diabetes (24).

However, the association between convenience stores and the prevalence of obesity and hypertension is not always consistent. Some studies have reported that convenience store density per capita is negatively associated with obesity prevalence in the United States (35), while others have found no significant association between convenience store density and hypertension in Mexico (36). These inconsistencies may reflect not only differences in study design but also differences in the role that convenience stores play within local food environments. In some neighborhoods of Chicago, convenience stores may function as important food sources within communities with limited food access (35). In contrast, convenience stores account for only a small proportion of the overall food retail environment in Mexico (36). South Korea differs from these settings in that it has a very high convenience store density, and convenience stores serve as readily accessible sources of ready-to-eat and processed foods. These contextual differences suggest that the extent to which convenience store density influences dietary behaviors and chronic disease risk may vary substantially across countries.

Unlike previous studies, our study applied spatial analysis to examine the association between convenience store density and chronic disease prevalence while accounting for spatial autocorrelation. After this adjustment, convenience store density remained significantly associated with hypertension, but not obesity. This finding suggests that geographic clustering may play an important role in explaining regional variation in chronic disease prevalence, particularly for obesity.

Environmental approaches that support healthy eating habits should be considered alongside concerns about the rapid increase in convenience stores. The spatial distribution and regional clustering patterns identified in this study provide insights into the geographic variations in convenience store density and obesity and hypertension prevalence in South Korea. Additionally, the spatial characteristics observed in regions with relatively high prevalence of obesity and hypertension offer important context for explaining regional differences in disease burden. Moreover, in areas with high convenience store density, factors, such as encouraging the availability of healthier food options, promoting low-sodium alternatives, and supporting nutrition education initiatives, should be considered in efforts to address chronic disease risk.

This study has some limitations. First, BMI was estimated from self-reported data, which often leads to height overestimation and weight underestimation (37), potentially resulting in an underestimated obesity prevalence. Second, the cross-sectional nature of our study makes it unsuitable for establishing causal relationships. Third, the density of convenience stores does not reflect their accessibility, frequency of use, or the consumption of convenience store food. Although people mainly visit convenience stores near their homes, considering only the local food environment may not fully reflect their actual usage of convenience stores (38). Fourth, this study was conducted at the district (ecological) level, and the observed associations reflect area-level rather than individual-level relationships. Residents of districts with high convenience store density are not necessarily the same individuals who frequently use convenience stores or who have obesity or hypertension, and these findings should not be interpreted as individual-level causal effects. Finally, this study focused solely on convenience store density without accounting for other environmental factors, such as the Retail Food Environment Index (RFEI), fast-food restaurant ratios, or park availability, which may be associated with the prevalence of chronic diseases (39–41). Future studies should consider these broader environmental influences to provide a more comprehensive understanding of the impact of convenience store density on chronic disease prevalence.

Despite these limitations, this study makes several important contributions. To our knowledge, it is one of the few studies to examine the association between convenience store density and chronic disease prevalence in South Korea using spatial analysis. By accounting for spatial autocorrelation and visualizing regional clustering patterns, this study provides a more appropriate assessment of the geographic relationship between the local food environment and chronic disease prevalence. In addition, the use of nationally representative survey data and adjustment for regional characteristics strengthen the reliability of the findings.

## 5 Conclusion

In summary, convenience store density was significantly associated with hypertension prevalence after accounting for spatial autocorrelation. These findings suggest that the local food environment may be relevant in understanding regional patterns of hyper-tension prevalence. From a policy perspective, efforts to improve the nutritional quality of foods available in convenience stores, promote lower-sodium and healthier meal options, and provide point-of-purchase nutrition guidance may help reduce chronic disease risk. Further research is needed to clarify how convenience store environments influence dietary quality and long-term cardiometabolic health.

## Data Availability

The raw data used in this study were provided through the KCHS website after obtaining approval from the KDCA.

## Footnotes

1
https://qgis.org/es/site/
