# Supplementary material for: Spatial analysis of the association between convenience store density and the prevalence of obesity and hypertension in South Korea
Source: Front Public Health. 2026 Jul 22;14:1857479. doi: 10.3389/fpubh.2026.1857479 (PMC13437698; doi:10.3389/fpubh.2026.1857479)
Supplement: Supplementary file 1 [file Supplementary_file_1.docx]

**Supplementary Figure S1.** Conceptual DAG of the convenience store density and obesity and hypertension prevalence pathways.

Dietary behaviors were considered a potential unmeasured pathway linking convenience store density to obesity and hypertension prevalence. Sociodemographic and lifestyle/health-related factors were included as potential confounders based on prior evidence and were adjusted for in the regression
