# Supplementary material for: Spatial analysis of the association between convenience store density and the prevalence of obesity and hypertension in South Korea
Source: Front Public Health. 2026 Jul 22;14:1857479. doi: 10.3389/fpubh.2026.1857479 (PMC13437698; doi:10.3389/fpubh.2026.1857479)
Supplement: Supplementary file 2 [file Supplementary_file_2.docx]

**Supplementary Figure S2.** Administrative regions of South Korea.

Administrative regions (17 provinces and metropolitan cities) of South Korea used for regional interpretation of district-level spatial analysis.
