# Supplementary material for: Spatial analysis of the association between convenience store density and the prevalence of obesity and hypertension in South Korea
Source: Front Public Health. 2026 Jul 22;14:1857479. doi: 10.3389/fpubh.2026.1857479 (PMC13437698; doi:10.3389/fpubh.2026.1857479)
Supplement: Supplementary file 3 [file Table_1.docx]

**Supplementary Table S1.** Sensitivity analysis using reduced OLS and SEM models for obesity and hypertension prevalence.

|  | Obesity prevalence | | |  | Hypertension prevalence | | |
| --- | --- | --- | --- | --- | --- | --- | --- |
| Variables | OLS^1)^ |  | SEM |  | OLS |  | SEM |
| Convenience store density | 1.012 (0.421)* |  | 0.701 (0.438) |  | 2.108 (0.418)*** |  | 1.100 (0.424)** |
| 20–30 age group (%) | 0.134 (0.048)** |  | 0.161 (0.050)** |  | -0.461 (0.048)*** |  | -0.506 (0.048)*** |
| Men (%) | 0.337 (0.132)* |  | 0.282 (0.129)* |  | -0.217 (0.131) |  | -0.137 (0.122) |
| High school graduate or lower (%) | 0.100 (0.031)** |  | 0.109 (0.031)*** |  | 0.256 (0.030)*** |  | 0.231 (0.030)*** |
| Unemployment (%) | -0.065 (0.037) |  | -0.081 (0.036)* |  | -0.064 (0.036) |  | -0.046 (0.034) |
| Current smoking (%) | 0.284 (0.079)*** |  | 0.228 (0.079)** |  | -0.256 (0.078)** |  | -0.169 (0.075)* |
| Individuals reporting subjective stress (%) | 0.039 (0.059) |  | 0.012 (0.056) |  | 0.169 (0.059)** |  | 0.101 (0.051)* |
| Lambda |  |  | 0.3200** |  |  |  | 0.4837*** |
| Moran's I | 0.1245 *** |  | -0.0104 |  | 0.2236 *** |  | -0.039396773 |
| R^2^ | 0.4179 |  |  |  | 0.8461 |  |  |
| Adjusted R^2^ | 0.4010 |  |  |  | 0.8417 |  |  |
| AIC | 1175.4 |  | 1167.0 |  | 1171.1 |  | 1142.1 |

^1)^values are presented as coefficient (standard errors)

**p* < 0.05*, **p* < 0.01, ****p* < 0.001.

Lambda (λ) in the Spatial Error Model (SEM) indicates the degree of spatial autocorrelation in the error terms. A significant and positive λ suggests that residuals in one region are spatially correlated with those in neighboring regions, highlighting the presence of spatial dependency that must be accounted for to avoid biased estimates.

OLS, Ordinary least squares; SEM, Spatial error model; and AIC, Akaike information criterion.
